# Implementation of World Health Organization Integrated Management of Childhood Illnesses (IMCI) Guidelines for the Assessment of Pneumonia in the Under 5s in Rural Malawi

**DOI:** 10.1371/journal.pone.0155830

**Published:** 2016-05-17

**Authors:** Ngozi Kalu, Norman Lufesi, Deborah Havens, Kevin Mortimer

**Affiliations:** 1 Department of Clinical Sciences, Liverpool School of Tropical Medicine, Liverpool, United Kingdom; 2 Ministry of Health, Lilongwe, Malawi; University of Southern California, UNITED STATES

## Abstract

The Cooking and Pneumonia Study (CAPS) is a pragmatic cluster-level randomized controlled trial of the effect of an advanced cookstove intervention on pneumonia in children under the age of 5 years (under 5s) in Malawi (www.capstudy.org). The primary outcome of the trial is the incidence of pneumonia during a two-year follow-up period, as diagnosed by healthcare providers who are using the World Health Organization (WHO) integrated management of childhood illnesses (IMCI) pneumonia assessment protocol and who are blinded to the trial arms. We evaluated the quality of pneumonia assessment in under 5s in this setting via a cross-sectional study of provider-patient encounters at nine outpatient clinics located within the catchment area of 150 village-level clusters enrolled in the trial across the two study locations of Chikhwawa and Karonga, Malawi, between May and June 2015 using the IMCI guidelines as a benchmark. Data were collected using a key equipment checklist, an IMCI pneumonia knowledge test, and a clinical evaluation checklist. The median number of key equipment items available was 6 (range 4 to 7) out of a possible 7. The median score on the IMCI pneumonia knowledge test among 23 clinicians was 75% (range 60% to 89%). Among a total of 176 consultations performed by 15 clinicians, a median of 9 (range 3 to 13) out of 13 clinical evaluation tasks were performed. Overall, the clinicians were adequately equipped for the assessment of sick children, had good knowledge of the IMCI guidelines, and conducted largely thorough clinical evaluations. We recommend the simple pragmatic approach to quality assurance described herein for similar studies conducted in challenging research settings.

## Background

Pneumonia is the single largest infectious cause of death globally in children under 5 years of age, accounting for 15% of all deaths in this age group [1]. The highest disease burden is in sub-Saharan Africa, home to the most vulnerable children from the poorest households [2,3]. The Malawi Ministry of Health has estimated that approximately 500/1,000 children under the age of 5 years were diagnosed with an acute respiratory infection during 2009/2010, with a 1.5 per 1,000 case fatality rate [4].

Household air pollution from the burning of biomass fuels for cooking, heating, and lighting in homes increases the risk of pneumonia in young children [5,6]. It is estimated that dirty-burning fuels are used for cooking in up to 95% of all households in Malawi, and exposure to smoke from these fuels is likely responsible for a substantial burden of childhood pneumonia in this setting [7,8,9,10].

The Cooking and Pneumonia Study (CAPS) is an MRC, Wellcome Trust, and DfID (Joint Global Health Trials Scheme)-funded pragmatic cluster randomized controlled trial of an advanced cookstove intervention to prevent pneumonia in children under 5 years of age (under 5s) in Malawi (www.capstudy.org). The primary outcome of the CAPS trial is the incidence of pneumonia in under 5s during a 2-year follow-up, as diagnosed by healthcare providers who are blinded to the trial arms and are using the WHO integrated management of childhood illness (IMCI) pneumonia assessment protocol [10]. The IMCI criteria were chosen because they represent a standardized diagnostic approach and it is the approach used for the diagnosis of pneumonia in Malawi where access to more sophisticated diagnostic methods (e.g. physician evaluation or chest X-ray) are not routinely available. Diagnostic evaluations for the majority of the CAPS participants are conducted at health facilities located in the study districts of Chikhwawa and Karonga. Here, we report the findings of a cross-sectional study that was performed to evaluate the practical implementation of the IMCI pneumonia protocol in health centers in the catchment area for the CAPS trial.

## Methods

### Setting

The study took place in the outpatient departments of health facilities located within the catchment area of the 150 village-level clusters enrolled in the CAPS trial across the two study locations of Chikhwawa and Karonga in Malawi between May and June 2015. The CAPS trial is being conducted in close partnership with the Malawi Ministry of Health and local health facilities have received support from the trial to achieve high quality clinical assessments and documentation of pneumonia diagnoses by 1) providing training for healthcare providers in the use of the IMCI pneumonia protocol, 2) including a summary of the IMCI pneumonia assessment protocol in each trial participant’s health record, and 3) providing diagnostic aids to the health centers (themometers, respiratory timers and pulse oximeters). The trial has also provided supplies of antibiotics to health centers for the treatment of pneumonia in children when local supplies have run short.

### Participants

Nine of a possible 11 health facilities, were selected for participation on the basis that these health facilities saw the highest numbers of CAPS patients according to monthly reports. The health facilities were a district (secondary referral) hospital, a rural (primary referral) hospital, 5 health centers, a private clinic and a village health post. Five were government health facilities, two Christian Health Association of Malawi (CHAM) health centers, one a Army Health Center, and one a private health center.

Outpatient care at the selected health facilities is primarily provided by clinical officers, clinical officer interns, medical assistants, medical assistant interns, registered nurses, nurse technicians, and health surveillance assistants [11,12]. All healthcare providers at work during the period of the study were eligible to participate provided they were involved with the provision of routine pediatric outpatient care and agreed to take part.

### Measurements

The first author (NK) observed consultations with under 5s at the participating clinics, during which they completed a checklist for the availability of 7 essential equipment items (health record, pen, stethoscope, respiratory timer, thermometer, symptom/sign/treatment aid, and pulse oximeter), a checklist of 13 clinical evaluation tasks (ask reason for clinic visit, ask child’s age, ask about cough or difficult breathing, ask about drinking/breastfeeding, ask about vomiting, ask about convulsions, check temperature, measure respiratory rate, examine for grunting/stridor/wheezing, examine for signs of respiratory distress, assess conscious level, perform pulse oximetry and checked for presence of fever or referred to temperature if taken previously), and administered a test of knowledge of the IMCI pneumonia guidelines. These assessment tools were developed from the CAPS trial protocol along with a validated generic health-facility survey tool formulated by the WHO and a knowledge test developed by USAID that had 12 multiple response questions with a maximum possible score of 65 [10,13,14].

### Statistical methods

Descriptive statistics are reported for all participating health facilities and clinicians.

These were computed using IBM SPSS version 22.

### Ethical considerations

This study was conducted in partnership with the Malawi Ministry of Health as part of the quality assurance for the CAPS trial, which has been approved by the Malawi College of Medicine Research Ethics Committee (Ref P.11/12/1308) and the Liverpool School of Tropical Medicine Research Ethics Committee (Ref 12.40). Written informed consent for participation in the CAPS trial was obtained firstly at community level and then at individual household level on behalf of the children enrolled in the trial. In addition, all clinicians and families provided verbal consent before being observed for the study.

## Results

Twenty-three clinicians were included in the study. All participants completed the IMCI pneumonia knowledge test, and 15 were observed while conducting a total of 176 patient consultations.

The child’s health record and a pen were available for 176 (100%) of consultations with stethoscope, respiratory timer, thermometer, symptom/sign/treatment aid, and pulse oximeter available at 159 (90%), 156 (89%), 152 (86%), 115 (65%), and 110 (63%) consultations respectively. All 7 of the essential items were available during 31% (54/176) of the patient-provider encounters. The median number of essential items available during all observed consultations was 6 (range 4 to 7).

Clinicians performed all 13 clinical evaluation tasks during 7% (12/176) of the encounters, and the median number of tasks performed was 9 (range 3 to 13). The least frequently performed tasks were inquiry regarding history of convulsion (17%; 30/176) and pulse oximetry (35%; 62/176). Decreased oxygen saturation and history of convulsion are regarded as signs of severe pneumonia in the version of the IMCI guidelines in current use in Malawi. Assessments for other indicators of pneumonia severity, including grunting/stridor/wheezing, signs of respiratory distress, and lethargy/unconsciousness were performed in 94% (166/176), 88% (155/176), and 98% (173/176) of visits, respectively. Inquiries regarding feeding and vomiting were made in 55% (96/176) and 47% (83/176) of the patient visits.

The median IMCI pneumonia knowledge score was 75% (range 60% to 89%).

## Discussion

In general, the clinicians in the catchment areas for the CAPS trial had access to essential equipment for the assessment of sick children, conducted largely thorough clinical evaluations, and had adequate knowledge of the IMCI guidelines.

The availability of essential equipment did not necessarily translate to use during the physical examination. For example, while a pulse oximeter was available at 63% of patient visits, it was only used during 35%. Respiratory timers, which were available at 89% of the observed visits, were only used in 60%, and thermometers, which were available for 86% of the visits, were only used during 68%. Diagnostic supports such as these are not mandatory for IMCI implementation, because case detection is based on history and physical examination rather than diagnostic testing. However, the availability of such supports can contribute to improved quality of care [15,16].

Most clinical evaluations included fewer than the 13 tasks we were looking for. This is to be expected since it is possible to make a correct diagnosis without all tasks being completed and in this, like many, health settings the most efficient route to diagnosis and treatment is usually taken. Having said this, it is notable that the group of clinicians who participated in the present study performed more thorough history and physical examinations than a group of clinicians who were observed during a previous evaluation of IMCI pneumonia care in Malawi, although areas of weaker performance were consistent with previous studies here and in other countries [17,18].

The measurement of signs and symptoms of severe pneumonia or other severe febrile disease, including convulsions, inability to feed, lethargy/unconsciousness, grunting, stridor, wheezing, obvious respiratory distress, and diminished oxygen saturation, varied. History taking tasks were the most infrequently performed components of the clinical assessments for severity of disease, with inquiry regarding history of convulsions completed in only 17% of the encounters. In terms of the physical examination, pulse oximetry was performed the least often.

Convulsions and reduced oxygen saturation have been associated with increased risk of mortality, and oxygen saturation has proven to be a practical and objective measure of severity of illness and a good predictor of pneumonia [19,20,21,22]. Bjornstad et al have also reported infrequent assessment of danger signs by clinical officers in Malawi [17], and studies evaluating IMCI assessments in other countries have reported similar findings [23,24]. However, in the present study, these danger signs were assessed in almost all of the children who presented with grunting at all of the participating health centers in this study, which indicates that danger signs are assessed when it is most clinically relevant.

Furthermore, the largest participating health facility in this study, the Chikhwawa District Hospital, where 40% of the observations were conducted, has implemented an emergency triage assessment treatment (ETAT) protocol. ETAT utilizes a "traffic light" system, in which non-urgent patients are given green cards, priority patients are given yellow cards, and emergency patients receive red cards and are sent straight to the emergency department. Implementation of ETAT has been associated with improved patient outcomes and reduced mortality [25,26]. Under 5s with severe pneumonia were rarely seen by the participating clinicians at the pediatric outpatient department during normal clinic hours at the Chikhwawa District Hospital, and this might also explain why these occasionally-encountered tasks were infrequently performed.

There was a greater number of clinicians and health facilities included in this study than in previous evaluations of IMCI protocols in Malawi. This allowed a more detailed description of clinicians’ performance during routine assessments of children presenting with cough or fever. The study by Bjornstad et al, evaluated the quality of IMCI-directed pneumonia care by clinical officers at a single government-operated district health facility in urban Malawi [17]. In contrast, our study included five teams of providers at four types of health facilities operated by government, non-profit organizations, and the private sector, in rural parts of Malawi.

The finding that clinicians at the health facilities included in our study had higher scores on IMCI knowledge tests and completed a greater number of assessment tasks in comparison to participants in previous evaluations of IMCI providers using similar measures [17,18,24] might reflect the continued support provided by the CAPS team at these facilities. Prior to the implementation of the CAPS trial, the study team provided the clinicians at participating health facilities in both districts with essential tools required for clinical assessment of pneumonia and with IMCI training. Refresher IMCI training was provided during the 3 months preceding this study, and IMCI protocols have been shown to improve the skills of health workers and are associated with reductions in child mortality [27,28,29].

One of the main limitations of this study is the unintentional impact that the presence of the observer may have had on the participating health workers. Another limitation is that the IMCI knowledge test was based on a previously-developed test and contained subjective questions. To address the latter issue, the questions were thoroughly evaluated for clarity of the English language and for standardization of the scoring system by the CAPS project manager, who is a physician in Malawi, along with two senior pediatricians, resulting in questions and answers that met best practice standards according to IMCI-based case management protocols in Malawi.

In conclusion, we believe that this study provides reassurance that the clinical assessment for pneumonia in under 5s in the areas where the CAPS trial is being conducted is of sufficient quality for the clinical care of young children with acute respiratory illness. It also provides reassurance that the on-the-ground clinical assessments are sufficient for the needs of the trial which is a pragmatic, real-life study implemented in an especially resource poor setting in one of the world’s poorest countries where health systems are particularly stretched. For the purposes of the trial we have included additional quality assurance measures including 100% Source Document Verification of all pneumonia diagnoses and review by an Independent Endpoint Review Committee which will be described separately. The simple and flexible approach to quality assurance of clinical assessments described herein can be recommended for similar studies conducted in challenging research settings.

## Supporting Information

S1 File Individual data points from which summary statistics were computed. (XLSX)
